# “Six months is a lot of time to lie”: Anticipated stigma, disclosure and treatment preferences among participants in a tuberculosis therapeutic trial

**DOI:** 10.21203/rs.3.rs-8277920/v1

**Published:** 2025-12-22

**Authors:** Faith Mugodhi, Cecilia Kanyama, Francis Makiya, John Metcalfe, Charity Potani, Kimberly K. Scarsi, Isabelle Weir, Jennifer Furin

**Affiliations:** University of Zimbabwe-Clinical Trials Research Centre (UZ-CTRC); University of North Carolina Project; Johns Hopkins Project, Blantyre Clinical Research Site; University of California, San Francisco (UCSF); University of North Carolina Project; University of Nebraska Medical Center; Harvard T.H. Chan School of Public Health; Harvard Medical School

**Keywords:** Tuberculosis, clofazimine, disclosure, stigma, hyperpigmentation, preferences

## Abstract

**Background:**

Tuberculosis (TB) is a leading cause of morbidity and mortality around the world. TB treatment is complex and clinical research has focused on shortening duration. Understanding patient preferences around treatment shortening is an important field of study, especially is shorter regimens contain drugs that have side effects that are challenging for people receiving treatment. Clofazimine, a drug that can cause skin hyperpigmentation has been proposed as a component of shorter TB treatment regimens. Concerns about hyperpigmentation and its impact on disclosure have been raised as issues that could make such treatment unacceptable.

**Methods:**

This was an exploratory, qualitative sub-study using in-depth interviews (IDIs) among purposively selected participants in the CLO-FAST trial (NCT04311502). CLO-FAST randomized participants to receive a standard, 6-month TB treatment regimen or a 3-month regimen containing clofazimine. A qualitative, exploratory sub-study of participants in the trial was undertaken to explore treatment preferences. Participants were asked about their experience with their assigned treatment regimen with a focus on disclosure of TB status and preferences for treatment. Data were analyzed by trained qualitative researchers using the theoretical framework of acceptability and the disclosure decision model.

**Results:**

Twenty-three participants from India, Malawi and Zimbabwe were selected from the 89 participants in the parent trial. The major themes that were generated were: 1) decisions around deliberate disclosure; 2) experiences with inadvertent disclosure; 3) consequences of inadvertent disclosure; and 4) the impact of inadvertent disclosure on regimen preferences. Most participants expressed a preference for shorter regimen even if it led to skin color changes. Preferences for a longer regimen seemed to be driven by anticipated concerns around inadvertent disclosure and by a belief that longer regimens were more potent.

**Conclusions:**

Preference assessment can be successfully built into therapeutic TB trials. Most participants prefer shorter treatment but concerns about potency and skin discoloration were expressed. Additional work is needed to delineate drivers of therapeutic preferences among people with TB.

**Trial Registration:**

NCT04311502, registered November 5, 2021.

## Background

Every year, ten million people around the world become newly sick with tuberculosis (TB), including 400,000 individuals with drug-resistant forms of disease (DR-TB)^1^. Treating TB is challenging, as multi-month courses of four to seven medications must be taken daily to achieve non-relapsing cure^2^. Despite being a treatable and preventable infection, TB kills more than 1.2 million people globally every year^3^.

The past decade has witnessed notable achievement in TB therapeutics, with new drugs developed or repurposed to target *M. tuberculosis*^4^. One of these repurposed agents is the riminophenazine agent clofazimine, which has been used to treat leprosy and DR-TB for decades. Preclinical studies suggest using clofazimine within first-line DS-TB treatment regimens could allow for significantly shortened treatment duration, making it more tolerable for people diagnosed with the disease^5^. Unfortunately, clofazimine is associated with skin hyperpigmentation which can persist for weeks to months after therapy has been completed^6^. Because TB is a highly stigmatized condition^7^, drugs that cause a change in appearance could have serious consequences for individuals who receive them, especially if they lead to inadvertent disclosure^8^. Disclosure is the process by which people share their illness diagnosis with others in their social circles, and when it is done as part of a deliberate decision, it can lead to increased social and community benefits^9^. Inadvertent disclosure happens when an individual’s health status becomes known to members of their social circles without the sick individual choosing to share that information^10^. This can occur for a number of reasons, including a change in physical appearance^11^. It is unknown how acceptable a shorter regimen containing clofazimine might be to people being offered such treatment or how it impacts the process of disclosure of TB status. We sought to determine the acceptability of such a regimen through exploration of participant experiences using qualitative methods^11^ as part of a multi-center trial assessing the efficacy and safety of a shorter, clofazimine-containing regimen for DS-TB.

## Methods

This was an exploratory, qualitative sub-study using in-depth interviews (IDIs) among selected participants in the CLO-FAST trial **(**NCT04311502, **registered November 5, 2021)**^12^. The Advancing Clinical Therapeutics Globally (ACTG) network launched the A5362 CLO-FAST trial in 2020. This phase 2C trial compared the 26-week standard of care regimen consisting of isoniazid, rifampicin, pyrazinamide, and ethambutol (standard of care or “SOC” arm) with a 13-week regimen consisting of clofazimine, rifapentine, isoniazid, pyrazinamide, and ethambutol (“CFZ” arm).

Participants at three sites (in India, Malawi, and Zimbabwe) were randomly selected to participate in an in-depth interview to determine their experience with the treatment regimen they received. Interviews were conducted by trained study staff after at least four weeks of TB treatment had been administered. Study staff explained the purpose of the interview before beginning the discussion. The questions asked during the interviews aimed to elicit participants’ experiences with disclosing their TB status and with taking the A5362 trial regimen to which they were assigned. Responses were captured with verbatim quotations in a case report form (CRF) by trained site investigators and analyzed at the end of the trial. The interview questions are included in Table 2 below. The interviews were not recorded, and supplementary field notes beyond the participant responses were not taken during the interviews. Interviews were done at the clinical research sites at each of the participating sites.

**Figure 1:**
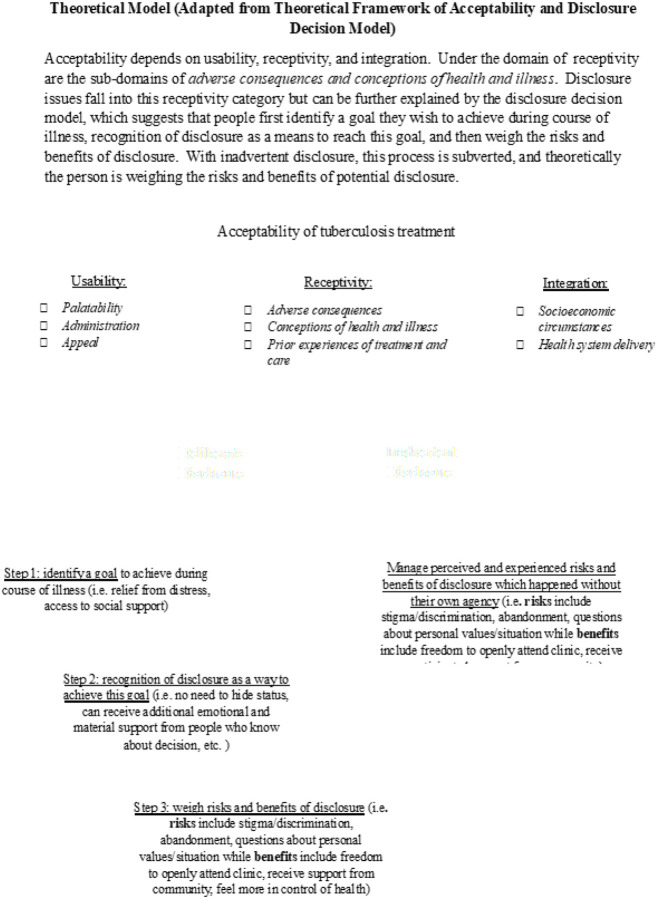
Theoretical model. Adapted from Theoretical framework of acceptability and Disclosure Decision model (Omarzu, 2000 and Wademan et.al, 2022)

Standard qualitative methods were used to analyze the responses for theme and content.^11^ The interview responses were coded by two experienced qualitative researchers employed by the ACTG (JF developed the initial codes which were then reviewed by F. Mugodhi) and then shared with the (sub) study team for further input. The theoretical models used in the analysis were the Theoretical Framework of Acceptability (TFA) developed for pediatric TB^13^ and the Disclosure Decision Model (DDM)^14^ used to guide HIV studies focused on disclosure. These two models were combined into a single analytic framework summarized in Fig. 1 below. Models were combined as both represented elements to guide acceptability and disclosure issues. The TFA focuses on domains that make up most factors which are relevant to the overall acceptability of TB treatment, which are usability, receptivity and integration while the DDM focuses on social rewards and goals that people may be focused on when weighing the risks and benefits of disclosure. Some major themes were identified before the start of the sub-study using the aims as guidance while others were generated during the coding exercise. The intended sample size was 40 participants, but this was not reached as the parent protocol was halted early by the protocol’s Data and Safety Monitoring Board. Data saturation was thus not considered in the analysis of the sub-study.

Researchers engaged in reflexive practice to reduce bias and maintain objectivity. Several potential sources of bias were identified, including the medical training of some of the research team, the fact that the trial was stopped early by the Data and Safety Monitoring Board, and the possibility that the CRFs limited the reporting of the participant responses. Whenever possible, the analysis team tried to account for these biases and limitations, which is further detailed in the limitations section of this paper. Participants did not review the study data, but findings from the study were shared with them following usual ACTG practices.

**The trial was registered with** ClinicalTrials.gov: NCT04311502. A central institutional review board and national and local ethics committees approved the protocol. The ACTG Global Community Advisory Board as well as local community advisory boards at recruiting sites provided input into, and approved, the protocol. The participants provided written consent. All methods/study procedures were carried out in accordance with the revised Declaration of Helsinki 2013 and 2024. All participants were informed about the purpose of the study, assured of confidentiality, and provided written consent prior to participation. Participation was voluntary, and respondents could withdraw at any time without consequence.

## Results

Twenty-three participants from India, Malawi and Zimbabwe were selected from the 89 participants in the parent trial. All 23 gave consent and participated in the IDIs. Demographics and questions asked in the interview are included in Tables 1 and 2 respectively.

A large number of the participants were between the ages of 26–35 while 69.6% were male. Treatment arms were almost equally divided with 12 being in the Clofazimine (CFZ) arm and 11 being in the standard of care (SOC) arm.

Of the participants, 22 were interviewed once and one participant was interviewed twice when she decided to leave the trial early in order to capture any significant changes to her earlier responses. However, her responses were internally consistent and thus only contributed once to the data analysis.

The major themes that were generated were: 1) decisions around deliberate disclosure; 2) experiences with inadvertent disclosure; 3) consequences of inadvertent disclosure; and 4) the impact of inadvertent disclosure on regimen preferences. Each of these is described in more detail below.

### Decisions around deliberate disclosure, goals and people chosen for disclosure

This theme describes what facilitated a person’s disclosure and if there were any goals that they were trying to reach. The theme also describes the nature of the relationship that existed between the participant and the person they disclosed to.

All participants reported that they had disclosed their TB diagnosis to at least one person. Often this person was a family member, but participants also reported disclosing to friends and co-workers. One motivation given for disclosing their TB diagnosis was to protect others, as shown in the quote below:

“Yes, I have told everyone at my work, my family and my friends because this disease is communicable, and I didn’t want them to have this disease so I have informed them.”

(female aged 33, CFZ arm)

Participants also reported disclosing so they could have support, as demonstrated in the quote below:

“I told my sisters and my brothers. Because they are the ones close to me who could offer me help.”

(male aged 30, SOC arm)

Participants also reported that they chose to disclose so they could avoid the negative consequences of having to hide their status, as described by one participant:

“Since I have to go for follow up and [directly observed therapy] DOT, I informed my co-workers and employer about my disease because 6 months is a lot of time to lie.”

(male aged 56, SOC arm)

Some participants reported disclosing to some people but not to others—i.e. selective disclosure—out of fear of experiencing negative consequences. As one participant reported:

“I told my parents and brothers. I did not feel like revealing to everyone because of people’s reactions, they would start talking about it everywhere, even with joy, pleased that I am sick.”

(male aged 29, SOC arm)

Some also reported disclosure was not always driven only by their choices/wishes. That is, they disclosed their TB status to individuals who already had suspicion about their TB. Sometimes disclosure was unavoidable due to close proximity and interaction of certain family members. From the participant’s reports, some members of their family were so close to the participants that they could notice changes in behavior and routine, prompting questions about change, as reported by one participant:

“Only my son-in-law and daughter know about my sickness. I told my daughter because she asked me why do I go to the hospital often and so I told her that I have a pulmonary tuberculosis infection. Also, my son-in-law was always there when we were visiting the clinic. So he knew what is my medical condition.”

(female aged 43, CFZ arm)

### Inadvertent/unintended disclosure

Inadvertent or unintended disclosure happens when the person’s TB status is disclosed to others without the person who has TB telling them. Most of the participants reported they were not worried about inadvertent disclosure, as illustrated in the quote below:

“Even if other people get to know about my disease I won’t be bothered. Why should people be feeling bad about me having a disease when I am the one caring for my family and people around me?”

(male aged 56, SOC arm)

Some reported they were not worried because their status had already been inadvertently disclosed and nothing negative had happened to them as a result.

“No, I don’t feel worried. I feel people might have got to know about my disease when I got admitted during my treatment but there was no difference in their behaviour, so I am not sure. My friend’s relative had this disease so she knew about it and she was very supportive. I myself used to stay indoors and keep distance from them. People used to ask my mother about my health, but my mother used to tell them that I am sick. She didn’t tell them about tuberculosis.”

(female aged 33, CFZ arm)

Some participants reported they feared inadvertent disclosure and felt they would be treated differently if people found out they had TB. This is illustrated in the quote from a participant below:

“Yes. Because if they know they could mock me. Mainly my neighbors.”

(male, aged 30, SOC arm)

Others reported fearing more serious/drastic forms of discrimination, as stated by this participant who feared people at work finding out:

“I am worried because they will fire me from the job and will not hire me again.”

(female aged 21, SOC arm)

Others reported that they were worried at the beginning of their treatment, but since they were getting better, they were no longer worried who found out. This is illustrated in the quotation below:

“Right now, I am not worried because I passed through the stage to be worried. This was during the first days when I got diagnosed, because I had fear and I was afraid of rejection from people.”

(male aged 29, SOC arm)

It is noteworthy that even among participants who stated they did not worry about inadvertent disclosure, most described withholding information on their TB status from certain groups of people. This complexity of the disclosure issue is illustrated in the quote below:

“I am not worried if people that I did not tell that I have TB find out that I have TB because they are not able to assist me with anything in my life. I did not tell my first cousins (children from his father’s brothers) because there isn’t a good relationship.”

(male aged 39, CFZ arm)

Although most participants said they were not worried about inadvertent disclosure, some (2/13; 15%) in the CFZ-containing regimen and many (8/10; 80%) in the standard of care arm worried that CFZ-related skin color changes could lead to disclosure, as illustrated in the participant quote below:

“My color is what I am worried about, my complexion changed. I hear my relatives saying, you are changing color, they say it is like you are being scorched, it is like these pills are scorching you.”

(male aged 39, CFZ arm)

Others also reported that the weight loss they experience with TB could lead to unintended disclosure. Finally, people reported that going to the DOT center or to the TB area of the hospital/clinic could also lead to inadvertent disclosure. These are illustrated in the participant quotes below:

“Yes [I worry about people finding out I have TB]. I have felt that because of my weight loss and skin color change and I am sick for a long [time].”

(female aged 33, CFZ arm)

“People got to know about my disease since I had to go to the hospital to take the DOT. People used to see me going but never talked to me, neither did I interrupt any of them. I used to go quickly take my medicines and come back as soon as possible.”

(male aged 56, SOC arm)

### Consequences of inadvertent disclosure

About one third of participants reported that there could be negative consequences of inadvertent disclosure, including stigma and discrimination. A sample quote illustrating this is included below:

“People have negative attitudes about those with TB and I think they would be negative towards me.”

(male aged 29, SOC arm)

A small number of participants reported that these consequences would be mitigated the longer they were on treatment, as illustrated below:

“People may think that they will contract TB from me and will not feel comfortable around me. However, these thoughts were more during my early treatment days, and I never used to mix with other people except my immediate family who knew about the disease.”

(male aged 32, SOC arm)

A small number of participants (4) reported that they had experienced consequences of inadvertent disclosure, including isolation, discrimination and “mocking”. Of these, 3 were in the arm containing clofazimine. A sample quote is provided below:

“Yes, I saw that my neighbors went inside their houses as soon as they saw me outside my house. I also felt this with my friends.”

(female aged 21, SOC arm)

### Treatment regimen preferences

Almost two thirds of the participants reported that they would prefer a shorter (3-month) regimen that caused skin changes compared with a 6-month regimen that did not cause skin changes. Some illustrative quotes from participants are included below:

“I [would]choose treatment for 3 months with my skin changing complexion because taking pills for 6 months is painful. Just being a person always on pills for a long time ends up not being ideal, it’s worrisome always taking pills.”

(male aged 39, CFZ arm)

“I would choose treatment for 3 months even though the skin would change because what would be important to me is the effectiveness of the drugs and getting better quickly.”

(female aged 27, CFZ arm)

It is important that a “shorter” regimen was not always seen as better regimen, and almost one third of the participants expressed a preference for a longer regimen. As one participant reported:

“I would choose the 6 months because I do not want any changes on my skin or appearance because even after treatment, the changes may still remain. This may affect you even where you go anywhere or look for a job.”

(male aged 29, SOC arm)

And another stated:

“I would prefer the 6-month treatment because the skin changes would make people question my health status. This would in turn probably lead to rejection and stigmatization from people I mix with in the community or at my workplace.”

(male aged 32, SOC arm)

While most who expressed preference for a longer regimen did so due to fears about skin color changes and fear of stigmatization, some participants felt a longer regimen would be more likely to cure them or would be more potent against TB, as illustrated below:

“I feel the longer treatment is better than the shorter treatment since shorter can be inadequate then and chances [are] that some infection still remains. I feel 6 months will cure completely and I will get cured too.”

(male aged 56, SOC arm)

Some expressed no preference and said they would take any regimen that would cure them, as noted below:

“I would have chosen any treatment that would heal me. What I want is healing

(male aged 33, CFZ arm)

Importantly, most of the people who reported that they would prefer a longer regimen did not actually receive clofazimine (i.e. they were assigned to the standard-of-care arm). This suggests an element of anticipated stigma^15^ which might have been mitigated by lived/actual experiences with skin color changes. This can be seen in the quotation below from a participant in the clofazimine arm:

“Three months treatment is very good because I can get well soon and start living with my children. Even if my color changes my priority is to get well soon. I feel very good about the fact that I can get well soon and keep my children closer to me.”

(female aged 29, CFZ arm)

Participants also expressed a preference for the regimen that they actually received, since they felt it was “working” for them. This is seen in the participant quote below:

“I would choose the 6 months regimen because it has helped me a lot and I have not experienced anything bad.”

(male aged 38, SOC arm)

## Discussion

This was a qualitative, exploratory sub-study of the A5362 trial which aimed to elicit participants’ experiences with disclosing their TB status and with taking the study regimen to which they were assigned. The sub-study drew on the Disclosure Decision Model by Omarzu^14^ and the Theoretical Framework of Acceptability by Wademan and colleagues^13^. The sub-study yielded several important findings, demonstrating the feasibility of including qualitative research within larger randomized TB therapeutic trials. Additionally, it provided insights into participants’ disclosure behaviours, the impact of treatment side effects, treatment preferences and the subsequent impact of anticipated stigma on these preferences. These findings offer a deeper understanding of the social dimensions of TB treatment, viewed through the lens of established theoretical frameworks, and compared to past research.

We found that most participants deliberately disclosed their TB status to family or friends. This deliberate disclosure was often motivated by the need for emotional and practical support during treatment. According to the Disclosure Decision Model^14^, individuals manage their disclosures strategically to control their social environments and achieve personal goals^15^. Our findings reflect this, showing that participants chose their disclosure recipients carefully, aiming to maximize support and minimize the risk of stigma. Participants often disclosed their TB status to those who were likely to provide emotional support and practical assistance, such as family members and close friends. This selective disclosure aligns with findings from Nagarajan et al., who reported that TB patients in Chennai predominantly disclosed their status to family members to receive emotional and resource support^16^. Our sub-study extends these findings by showing that the decision to disclose is not only about receiving support but also about protecting others from potential infection, reflecting a sense of responsibility and care towards family and close friends. Moreover, participants would also disclose in order to avoid having to lie to their loved ones for the duration of their treatment phase. This strategic approach to disclosure illustrates how patients navigate their social environments to ensure both their well-being and that of their loved ones.

Inadvertent disclosure emerged as a concern among some of the participants in the clofazimine-containing arm who experienced visible side effects such as skin discolouration and among those who were in the standard of care arm who anticipated colour changes could lead to disclosure. Although we focused on appearance changes in our study, the skin hyperpigmentation was not the only issue mentioned by participants as being associated with inadvertent disclosure. Some were worried that their presence at a TB clinic, or a hospital area associated with TB care would be enough to reveal that they had TB. Our study was not designed to determine which activities or changes might be most worrisome to participants, but it is important to note that there are multiple aspects of care that were problematic in terms of inadvertent disclosure. Dormechele and colleagues found that the intention to conceal TB status was influenced by fear of stigma and social rejection, which is consistent with our findings^17^. Participants expressed concerns about how visible side effects, such as significant weight loss could lead to inadvertent disclosure and the consequent stigma. This fear of stigma is not unfounded, as illustrated by Wademan et al. in their study on the acceptability of TB treatments in children, where visible side effects were a significant concern for caregivers^13^. Our sub-study highlights the need for TB treatments that minimize visible side effects to reduce the risk of unintended disclosure and associated stigma. The experiences of our participants echo the findings of Nezenega et al., who reported that visible side effects and the social stigma associated with them were major factors influencing patient adherence to TB treatment in Ethiopia^18^. The fear of social rejection and discrimination due to visible treatment side effects underscores the importance of developing TB treatments that are both effective and socially acceptable.

The consequences of inadvertent disclosure, as reported by participants, included social isolation and stigma. This aligns with the findings of Nagarajan et al., who noted that TB patients often face varying degrees of social support and stigma following disclosure^16^. The fear of negative social consequences can significantly impact a patient’s mental health and adherence to treatment. Participants in our sub-study who experienced inadvertent disclosure reported feeling socially isolated and stigmatized. Some feared outright discrimination, such as losing their jobs or being ostracized by their communities. These findings echo those of Dormechele et al., who found that TB status concealment is often driven by a desire to avoid such negative outcomes^17^. Addressing these issues requires community education and supportive interventions that reduce stigma and promote understanding of TB. For instance, public health campaigns aimed at educating communities about TB and reducing potential sources of stigma could help alleviate some of the fears associated with inadvertent disclosure. Additionally, providing psychological support and counselling for participants in TB trials or for TB patients can help them cope with the emotional impact of stigma and social isolation.

The sub-study revealed a split in treatment regimen preferences among participants. Some preferred a shorter, more intensive regimen despite the risk of side effects, while others favoured a longer regimen to avoid visible changes in appearance and because they felt it was more effective. This finding highlights the need to balance medical efficacy with the potential for social repercussions. Participants who preferred the shorter regimen often cited the desire to return to their normal roles more quickly and reduce the daily burden of taking multiple pills. This preference for a shorter treatment duration aligns with the findings of Wademan et al., who reported that caregivers preferred dispersible tablet formulations for children due to their ease of use and shorter treatment duration^19^. On the other hand, participants who preferred the longer regimen were concerned about the visible side effects of clofazimine, such as skin discolouration. Data from our sub-study reveal that some participants who preferred the longer regimen were driven to this choice by a fear of stigma: those who reported preferring a longer regimen to avoid skin changes did not actually take the regimen with clofazimine. This concern reflects an anticipatory stigma, where individuals fear negative social consequences even if they have not yet experienced them. Stadler et al. noted that while shorter regimens are appealing, the side effects can significantly influence patient preference and perceived treatment acceptability^20^. Our findings suggest that while some patients prioritize the convenience and potential efficacy of shorter regimens, others are more concerned about the social implications of visible side effects, highlighting the need for personalized treatment approaches.

Our findings align with and extend existing research on TB treatment preferences and disclosure behaviours. The Disclosure Decision Model provides a useful framework for understanding the strategic nature of disclosure decisions^14^. The model posits that individuals evaluate the subjective utility and risk of disclosing their health status, which influences the content, depth, and duration of disclosure. Our sub-study supports this model by showing that participants carefully chose their disclosure recipients to maximize support and minimize stigma. The findings from Wademan et al. on the acceptability of TB treatments in children highlight the importance of considering the end-user’s perception and the context of treatment^19^. Our sub-study extends this by showing that adults also consider these factors when choosing their treatment regimens. The concerns about visible side effects and their impact on social interactions underscore the need for treatments that are not only medically effective but also socially acceptable. Dormechele et al., and Nagarajan et al., both highlight the significant role of stigma in shaping TB patients’ disclosure behaviours and treatment experiences^16, 17^. Our sub-study adds to this by showing that anticipated stigma, stigma and fear of social rejection influence not only disclosure decisions but also treatment preferences. This highlights the need for comprehensive interventions that address both the medical and social aspects of TB treatment.

One final finding of this sub-study is the feasibility of embedding qualitative research within larger TB therapeutic trials. Qualitative methods, such as the IDIs in this study provide rich, detailed data that can contextualize the quantitative findings. The use of IDIs allowed us to capture the detailed experiences and preferences of participants, offering deeper insights into their treatment journeys. The successful integration of qualitative research aligns with the broader movement in health research towards mixed methods approaches^21^, which combine the statistical power of quantitative data with the contextual richness of qualitative data. This combination is essential for developing a comprehensive understanding of participants’ as well as future patients’ experiences and outcomes. Our sub-study contributes to this movement towards mixed methods approaches by showing that qualitative research can be effectively conducted alongside rigorous clinical trials, providing valuable context and depth to the findings. For example, the nuanced narratives collected through IDIs in our sub-study revealed the complexity of participants’ behavioural responses to TB treatment and their strategies for coping with the stigma associated with the disease, insights that would be difficult to capture through quantitative methods alone.

## Limitations

This sub-study had several limitations that may have impacted its findings and generalizability. Firstly, the research was conducted across only three sites, limiting the diversity of the sample and potentially overlooking regional variations in TB treatment experiences and social contexts. The chosen sites in India, Malawi, and Zimbabwe may not fully represent the broader spectrum of cultural and socioeconomic conditions affecting TB patients worldwide. Secondly, for budgetary and logistical reasons, the interviews were not recorded and transcribed, which could have led to incomplete or inaccurate data capture. Without audio recordings, researchers relied on written notes and memory, which might not have captured the full depth and nuance of participants’ responses. This limitation could have affected the richness and accuracy of the qualitative data. However, the use of experienced and trained interviewers may have reduced this risk. Lastly, the A5362 trial was halted earlier than planned by the Data and Safety Monitoring Board although neither interviewers nor participants were aware of this decision during data collection. The early termination of the A5362 trial resulted in a smaller sample size and possibly limited the exploration of themes that might have emerged with a larger and more diverse participant pool. Despite these constraints, the findings provide valuable insights into TB treatment preferences, the impact of anticipated stigma of treatment preferences and the social dynamics of disclosure, highlighting areas for further research and intervention.

## Conclusion

People with TB enrolled in A5362 expressed different preferences regarding their treatment. Most people reported that they would prefer a shorter regimen lasting 3 months compared with a longer regimen lasting 6 months. Although therapeutic duration was an important factor in determining what participants preferred, anticipated stigma due to inadvertent disclosure and concerns about regimen potency were also reported by participants as reasons for preferring a longer treatment. Qualitative research documenting the experiences of people on TB treatment can and should be embedded in TB therapeutic trials to better describe the experiences of people enrolled in such research. Such data could provide important insights to support programmatic use of novel TB regimens.

## Supplementary Material

Supplementary Files

This is a list of supplementary files associated with this preprint. Click to download.

- COREQChecklist20.pdf
- Appendix.docx

## Figures and Tables

**Table 1:** Participant’s demographics

Variable	N (%)
*Age*	Range: 21–56 years
	21–25: 2 (8.7%)
	26–35: 11 (47.8%)
	36–45: 8 (34.8%)
	46 and above: 2 (8.7%)
*Sex*	F: 7 (30.4%)
	M: 16 (69.6%)
*Gender*	F: 7 (30.4%)
	M: 16 (69.6%)
*Treatment arm*	3-month clofazimine: 12 (52.2%)
	6-month standard of care: 11 (47.8%)

**Table 2:** Questions asked in interviews

1. Who have you told that you have been diagnosed with tuberculosis? Why did you tell these people?
2. Do you worry about people you did not tell that you have been diagnosed with TB finding out that you have TB? Why? Are there any people you are most worried might find out you have TB?
3. Is there anything about the treatment you have been taking that you worry might identify you as someone who has TB? If so, please describe:
4. What do you think might happen to you if people you did not tell you have tuberculosis find out that you have the disease?
5. Has anything bad ever happened to you when people have found out you have tuberculosis? If yes, please describe
6. If you had to choose between taking medicines for TB for 6 months with a treatment that caused no changes in your appearance OR taking a treatment for months that might cause your skin to change, which would you prefer? Why?
7. Is there anything else you would like to tell us about your experience being diagnosed and treated for TB?

## Data Availability

The datasets used and/or analysed during the current study are available from the corresponding author on reasonable request.

## Abbreviations

ACTG: Advancing Clinical Therapeutics Globally
CFZ: clofazimine
DDM: Disclosure Decision Model
DOT: Directly Observered Therapy
DR: Drug Resistant
DS: Drug Susceptible
HIV: Human Immunodeficiency Virus
SOC: Standard of Care
TB: tuberculosis
TFA: Theoretical Framework of Acceptability
